# Decentralized Clinical Trials in the Development of Drugs and Biological Products

**DOI:** 10.1007/s43441-023-00612-3

**Published:** 2024-02-02

**Authors:** Ryan Robinson, Leonard Sacks

**Affiliations:** https://ror.org/00yf3tm42 grid.483500.a 0000 0001 2154 2448 U.S. Food and Drug Administration, Center for Drug Evaluation and Research, Office of Medical Policy, Silver Spring, MD USA

**Keywords:** Decentralized clinical trial, Local health care provider, Digital health technologies, FDA, Remote

## Abstract

Decentralized clinical trials (DCTs) are trials where some or all of the trial-related activities occur at locations other than traditional clinical trial sites. FDA supports decentralization to improve participation in clinical trials. While there are benefits of DCTs, including convenience for participants, sponsors and investigators should be aware of potential challenges such as coordination of trial activities at locations other than traditional trial sites and supervision of delegated trial-activities performed remotely. Appropriate training, oversight, and up-front risk assessment and management will be key to implementing a DCT successfully.

## Commentary

Traditional clinical trials rely on a physical site where trial participants report, and where trial-related activities take place. Depending on the trial design, visits may be scheduled on a frequent basis and may continue for months or even years depending on the scientific question to be addressed. For participants this approach may be burdensome. Many challenges, including personal, physical, cognitive, and economic, may prevent willing individuals from participating. For example, it may be difficult for workers to take time off from their jobs, some individuals may have physical disabilities which limit their ability to travel, or some individuals may lack means of transportation to a trial site. The distance trial participants may need to travel to research sites is another potential deterrent to participation. In a 2018 study of 1600 clinical trial participants, the median distance traveled to a trial site was 25.8 miles, and participants from lower-income areas traveled further (58.3 miles vs. 17.8 miles) [1]. Many of these challenges are compounded in communities of lower socioeconomic status.

“Decentralized clinical trials (DCTs) are trials where some or all of the trial-related activities occur at locations other than traditional clinical trial sites” [2]. These trial-related activities may occur at locations such as a participant’s home or a local health care facility and may increase the convenience of trial participation.

“Advances in technology, including sensors, general-purpose computing platforms, and methods for data processing, transmission, and storage have” helped support the decentralization of trials and limit in-person visits [3]. Participants may attend scheduled visits virtually through telehealth which “use[s] electronic information and telecommunications technologies”, such as phone calls and videoconferencing, to support remote clinical health care [2]. In addition, digital health technologies (DHTs), such as wearables to track activity or mobile applications may be used to acquire data remotely from participants.

Use of existing community health care services, which includes many qualified providers who have not traditionally been used in the conduct of clinical trials, can also limit the burden of travel for trial participants. Such local health care providers (HCPs) can be utilized by investigators and sponsors in DCTs to perform trial-related activities which they are otherwise qualified to perform as part of their routine clinical practice. The use of local health care providers and local medical facilities close to participants’ homes can reduce the time required for trial participation. The use of local HCPs “may [also] improve engagement, recruitment, and retention of diverse participants (e.g., race, ethnicity, age, sex, and geographic location)” as well as “reduce cultural or linguistic barriers to participation” [2].

While the COVID 19 pandemic was a major incentive to use decentralized trial elements to sustain trials while avoiding in-person contact, the incorporation of decentralized elements into clinical trials is not a novel or recent concept. Elements such as the use of patient diaries and telephone follow-up or follow-up by mail have been used in trials for many years. In outpatient trials many activities are essentially decentralized once the participant leaves the trial site. For instance, participants are often provided medication that they must administer to themselves at home. Also, if they experience adverse events while away from the trial site, they must respond to and report these adverse events just as a participant in a decentralized trial would.

The first fully decentralized trial, with no in-person interaction, conducted under an investigational new drug application was the REMOTE trial in 2014 [4]. The trial was designed to investigate the effect of tolterodine on symptoms of overactive bladder (OAB). Using a web-based trial design, participation was solicited on the internet. Women with symptoms of OAB were randomized to tolterodine or placebo delivered by mail to patients’ homes. Informed consent was obtained electronically, and the trial endpoint was an eDiary documenting frequency of micturition. The initiative demonstrated the power of the internet to reach trial participants with 7230 completing the trial account registration page. However, only 18 participants were randomized and received treatment, 17 were included in the efficacy analysis, and 16 completed the trial. This was partly explained by the demanding requirements of the trial that included preliminary laboratory testing, complicated enrollment procedures and eligibility requirements, frequent reporting during a placebo run-in phase, and a 12-week intervention phase. Subsequent decentralized trials have often taken a more cautious approach, simplified trial-related procedures, and seen less attrition. For example, in a double-blind, fully remote 2020 trial comparing fluvoxamine with placebo in adult outpatients with symptomatic COVID-19, of 1337 adults with presumed or known COVID-19 assessed for eligibility, 181 were randomized, 152 received treatment as randomized and were included in the analysis, and 115 completed the trial [5]. In this trial, exclusion criteria were limited, the intervention lasted only 15 days, and participants self-reported their data (e.g. symptoms, physiological parameters, adverse events) via short twice daily surveys.

FDA has been supportive of trial decentralization, including the implementation during the COVID-19 pandemic to sustain clinical trials where in-person contact was not allowed. To this end the FDA published, the *Conduct of Clinical Trials of Medical Products During the COVID-19 Public Health Emergency* (March 2020, updated August 2021) [6]. This guidance provided recommendations on topics such as the conduct of remote (virtual) visits, delivery of low-risk investigational products to participants’ homes, and shipping of investigation products to local providers.

Outside of the setting of a pandemic, FDA has also supported decentralization and sees decentralization as a means by which to increase participant diversity and improve participation based upon convenience. This may be critical for drug development in rare diseases where patients are geographically dispersed or for patients who are physically or cognitively challenged, making frequent travel infeasible.

In December 2021, FDA published a draft guidance *Digital Health Technologies for Remote Data Acquisition in Clinical Investigations* which may facilitate the decentralization of trial-related activities by allowing data acquisition directly from participants. In May 2023, FDA also published a draft guidance *Decentralized Clinical Trials for Drugs, Biologics, and Devices* which includes recommendations on the design of DCTs, conduct of remote clinical trial visits and activities, the roles of sponsors and investigators in DCTs, investigational product considerations in DCTs, and safety monitoring in DCTs.

Despite the benefits of decentralized trials, there are challenges. For example, “coordination of trial activities with individuals and facilities in multiple locations that are not traditional trial sites” will often be required [2]. Also, sponsors and investigators must ensure supervision of those delegated to perform trial-related activities, such as local HCPs performing physical exams or providing drug infusion services. To manage such challenges, DCTs “generally include specific plans to facilitate decentralization of the trial” [2]. These plans include, as appropriate, details and processes for “the use of local health care facilities, local HCPs, and local clinical laboratory facilities; visits to trial participants’ homes; and direct distribution of the investigational product (IP) to trial participants at their locations”. In addition, a safety monitoring plan should be in place to “ensure that adverse events are appropriately captured and adequately addressed” and that participants know how “to respond to and report adverse events”, “where to seek medical assistance locally when necessary”, and how “to contact trial personnel to report adverse events and have pertinent questions answered” [2].

Whether trial decentralization is appropriate must be considered when initiating trial design. This decision should most importantly be based upon the assurance of the safety of trial participants and should take the nature of the IP into account. “Fully decentralized trials may be appropriate for drugs that are simple to administer or use, have well-characterized safety profiles, and do not require complex medical assessments” [2]. “Hybrid decentralized trials may be more appropriate in cases where the administration of an investigational drug or a complex medical assessment needs to be performed at a clinical trial site and some follow-up assessments could be performed remotely” as appropriate. In addition, “drugs best suited for direct shipment to participants’ homes or local HCPs include those with long shelf lives and those with good stability profiles”.

DCTs, whether fully decentralized or a hybrid of remote and in-person, are becoming more common throughout the clinical trial enterprise. FDA is supportive of trial decentralization with the hope that such decentralization will increase trial diversity as well as generally improve participation in clinical trials. While the benefits of DCTs, including improved convenience for participants, are apparent, sponsors should be aware of the potential challenges of conducting a DCT as well as understand which trials are appropriate to be conducted in a decentralized fashion. “Appropriate training, oversight, and up-front risk assessment and management will be key to implementing a DCT successfully” [2].
